# WHODAS 2.0 to Assess Disability in the Rehabilitation of Individuals With Chronic Respiratory Conditions: Responsiveness and Interpretability

**DOI:** 10.1002/pri.70170

**Published:** 2026-02-07

**Authors:** Chayenne Chylld César Lopes, Vanessa Garcia de Lima, Magno F. Formiga, Rafael Mesquita

**Affiliations:** ^1^ Department of Physiotherapy Federal University of Ceara (UFC) Fortaleza Ceará Brazil; ^2^ Masters Programme in Physiotherapy and Functioning Federal University of Ceara Fortaleza Ceará Brazil; ^3^ Graduate Programme in Cardiovascular Sciences Federal University of Ceara Fortaleza Ceará Brazil

**Keywords:** health of the disabled, patient reported outcome measures, rehabilitation, respiratory tract diseases

## Abstract

**Background and Purpose:**

Individuals with chronic respiratory conditions have physical impairments that lead to disability, but which can be alleviated with physical exercise. We aimed to examine the responsiveness and interpretability of the World Health Organisation Disability Assessment Schedule (WHODAS) 2.0 following an exercise‐based rehabilitation programme in people with chronic respiratory conditions.

**Methods:**

Observational cohort study with individuals who participated in an 8‐week rehabilitation programme with physical exercises. Before and after the programme, disability (12 questions WHODAS 2.0) and quality of life (Saint George's Respiratory Questionnaire—SGRQ) were assessed among other characteristics. Anchors‐ and distribution‐based methods were used to investigate a minimal important difference (MID).

**Results:**

33 participants were included (mean age of 59 ± 17 years, 67% female, 61% with chronic obstructive pulmonary disease). There was a reduction in the mean WHODAS 2.0 summary score after rehabilitation (mean difference −9.49, 95% CI ‐13.90 to −5.08; *p* < 0.001). A reduction in SGRQ total score was also observed (−15.5, 95% CI ‐21.7 to −9.7; *p* < 0.001). A regular correlation between the changes in WHODAS 2.0 and SGRQ was observed (*r*_s_ = 0.36; *p* = 0.04). Using anchor‐ and distribution‐based methods, the MID estimate varied between −6.22 and −5.02.

**Discussion:**

WHODAS 2.0, a standardised and easy‐to‐use questionnaire developed by the World Health Organisation (WHO), proved to be a responsive tool after an exercise rehabilitation programme in individuals with chronic respiratory conditions, and a reduction of at least 6.22 was considered clinically important.

## 1 Introduction

Individuals with chronic respiratory conditions, such as chronic obstructive pulmonary disease (COPD), asthma, and post‐COVID‐19 condition, experience impairments in functioning that lead to disability (GBD 2015 Chronic Respiratory Disease Collaborators 2017; Higginson et al. 2022). Dyspnoea is one of the most limiting symptoms in these conditions (Higginson et al. 2022; World Health Organizaton 2019), contributing to exercise intolerance and restrictions in activities of daily living, such as walking, compromising overall functioning (Global Initiative for Chronic Obstructive Lung Disease 2023; Global Initiative for Asthma 2023).

According to the International Classification of Functioning, Disability and Health (ICF), functioning encompasses body functions and structures, activities, and participation in society (World Health Organizaton 2019). In contrast, disability refers to impairments in body functions and structures, activity limitations, and/or restrictions in social participation (World Health Organizaton 2019). To assess disability, the World Health Organisation (WHO) developed the World Health Organisation Disability Assessment Schedule (WHODAS) 2.0, a generic instrument derived from a set of ICF codes (Global Initiative for Chronic Obstructive Lung Disease 2023; Üstün et al. 2010). WHODAS 2.0 is one of the few instruments grounded in a biopsychosocial model, integrating multiple dimensions of health (Üstün et al. 2010). Its clinical relevance has been demonstrated across diverse patient populations; for example, higher WHODAS 2.0 scores have been associated with medium‐term mortality in individuals with chronic diseases, including COPD, heart failure, and stroke (De Pedro‐Cuesta et al. 2013), as well as with postoperative mortality in older adults undergoing elective surgery (D. F. T. Lima et al. 2019).

Although WHODAS 2.0 has been used in individuals with chronic respiratory conditions (Federici et al. 2017), its responsiveness to commonly applied interventions in this patient population—such as exercise‐based rehabilitation programmes, including pulmonary rehabilitation (McCarthy et al. 2015; Osadnik et al. 2022; Pouliopoulou et al. 2023)—has not yet been investigated. Therefore, the aim of this study was to examine the responsiveness and interpretability of WHODAS 2.0 following an exercise‐based rehabilitation programme in individuals with chronic respiratory conditions. We hypothesised that WHODAS 2.0 would be responsive to the intervention and that a minimal important difference (MID) could be identified.

## 2 Methods

### 2.1 Study Design and Participants

Prospective observational cohort study carried out with individuals with chronic respiratory conditions recruited to participate in a rehabilitation programme with physical exercises offered by an extension project from a public university in Fortaleza‐CE, Brazil, between May 2022 and April 2024. These individuals were referred by pulmonologists or self‐referred. The programme was run in the university hospital's rehabilitation unit of the same university. Individuals were included if: (1) aged > 18 years; (2) had a confirmed diagnosis of a respiratory health condition (e.g., COPD, asthma, bronchiectasis, post‐COVID‐19 condition); (3) had symptoms that limited their daily activities; (4) were able to walk and participate in the proposed rehabilitation program; and (5) were able to provide informed consent. We excluded those who: (1) reported as the main limiting symptoms not directly related to the respiratory health condition (e.g., knee pain); (2) participated in a regular physical exercise programme (at least 30 min/day, 3x/week, for at least 3 months) in the previous year, and/or; (3) had health conditions that could make physical exercise unfeasible or contraindicated (e.g., unstable cardiovascular disease). This study was approved by a research ethics committee (approval number: XXX), and all participants provided informed consent.

This manuscript was prepared in accordance with the recommendations of the Strengthening the Reporting of Observational Studies in Epidemiology (STROBE) initiative (Von Elm et al. 2008) and Consensus‐based Standards for the selection of Health Measurement Instruments (COSMIN) reporting guideline for studies on measuring properties of patient‐reported outcome measures (Gagnier et al. 2021). Individuals with pre‐ and post‐rehabilitation data for the outcome of interest (i.e., WHODAS 2.0 summary score) were included in the current analysis. We aimed to recruit at least 30 individuals according to the COSMIN checklist (Mokkink et al. 2010).

### 2.2 Assessments

Sociodemographic and clinical data (e.g., age, sex, body mass index), lung function (spirometry) (Pereira et al. 2007; Graham et al. 2019), impact of dyspnoea on daily life (modified version of the Medical Research Council—mMRC scale) (Kovelis et al. 2008), functional exercise capacity (6‐min step test—6MST) (Salles Albuquerque et al. 2022), health‐related quality of life (HRQoL) (Saint George's Respiratory Questionnaire—SGRQ) (Camelier et al. 2006; Sousa et al. 2000), and disability (WHODAS 2.0) (Castro et al. 2015) were assessed before and after rehabilitation by trained researchers, following standard operating procedures. Furthermore, at the end of the rehabilitation program, a global change rating scale was used to verify the participant's perceived change in their degree of disability (Revicki et al. 2008).

SGRQ is a questionnaire already translated and validated into Brazilian Portuguese (Camelier et al. 2006; Sousa et al. 2000). It consists of 50 items distributed into three domains: symptoms (8 items), activities (16 items) and psychosocial impacts (26 items). A final score can be given for each domain, as well as a total score. Each of these scores can range from 0 to 100, with higher values representing worse HRQoL. A reduction of at least 4 points was adopted as the MID (Jones 2005). Originally, SGRQ was proposed for COPD and asthma (Jones et al. 1991); however, it has been used with different patient groups, such as in idiopathic pulmonary fibrosis (Cox et al. 2020), bronchiectasis (Spinou et al. 2016), and even individuals with COVID‐19 (Daher et al. 2020).

WHODAS 2.0 is a generic questionnaire that has also been translated and validated into Brazilian Portuguese (Castro et al. 2015). In our study, the 12‐item version was used, which represents six life domains: cognition, mobility, self‐care, getting along, life activities, and participation. Each question scores the degree of difficulty due to the participant's health condition in relation to 30 days before the assessment. The response items are: 0 (none), 1 (mild), 2 (moderate), 3 (severe), or 4 (extreme or unable to do it), but the option not applicable (N/A) can also be used when the question does not apply to the individual's reality. At the end, the scores are added up and corrected to the maximum possible value, thus generating a summary score that varies between 0 and 100, in which the higher the score, the greater the disability (Castro et al. 2015).

At the end of the rehabilitation programme, each participant was asked how they would classify their disability after participating in the programme, using a global rating scale of change (Revicki et al. 2008). It should be noted that, when asked about disability, the participant should understand both the physical aspects of his/her body, but also the activities he/she performed in his/her daily life and his/her participation in society. The response options were: (1) better, (2) slightly better, (3) about the same, (4) slightly worse, or; (5) worse.

### 2.3 Rehabilitation Programme

The rehabilitation programme with physical exercises included individualised and weekly progressed aerobic and peripheral muscle strength training, 2x/week supervised by trained physiotherapists plus 1x/week unsupervised at home, lasting 8 weeks (i.e., 16 supervised sessions in total), following international recommendations (Spruit et al. 2013). Aerobic training was performed on a treadmill or cycle ergometer (the former was preferred unless the participant had difficulties with it), starting with 10 min and progressing up to 30 min, setting the speed on the treadmill or the load in watts on the cycle ergometer (instructed speed around 50–60 rpm) to reach moderate intensity (3–6 on 0–10 Borg scale). Strength training was performed for both the upper and lower body, starting with two sets of 10 repetitions of the maximum tolerated load for at least 10 repetitions and progressing to three sets of 15 repetitions, always aiming to reach moderate intensity (11–13 on 6‐20 Borg scale). Strength training exercises included dumbbell standing shoulder press starting with the dumbbells in front of the thighs, bent‐over row with dumbbells, standing plantar flexion, and sit‐to‐stands. Breathing exercises and calisthenics were used for warming‐up, and stretching was used for cooling‐down. Balance exercises were performed by elderly individuals or those who had balance problems. Participants who were unable to adhere to the prescribed exercises or exercise intensity for any reason were excluded. Programme completion was defined as attendance at a minimum of 75% of supervised sessions (i.e., 12 sessions) and completion of the end‐of‐rehabilitation assessment. Participants who failed to meet these criteria were excluded from the analysis.

### 2.4 Statistical Analysis

Data were expressed as absolute and relative frequency, mean ± standard deviation (SD), or mean (95% confidence interval—CI). The Wilcoxon test was used to compare pre‐ and post‐rehabilitation variables, while the Spearman correlation coefficient was used for the correlation between the change in WHODAS 2.0 summary score and other variables. We included only participants for which we had no missing data on the variables of interest (complete‐case analysis). The responsiveness and interpretability analyses of WHODAS 2.0 were similar to those carried out by others (Nolan et al. 2019).

In order to evaluate responsiveness, we aimed to carry out the following analyses: 1^st^, the comparison of WHODAS summary score between pre‐ and post‐rehabilitation, and; 2^nd^, the comparison of the change in WHODAS summary score between those who did and those who did not had a clinically important improvement in SGRQ total score (≤ −4 points) (Jones 2005). To evaluate interpretability with the aim of proposing a MID estimate for the WHODAS 2.0 summary score, we performed anchor‐ and distribution‐based approaches. For the anchor‐based analysis, we first investigated the correlation between the change in WHODAS 2.0 summary score and the change in SGRQ total score, expecting at least a fair correlation (i.e., correlation coefficient ≥ 0.3) (Revicki et al. 2008). If this was observed, a linear regression analysis was run to estimate the change in WHODAS 2.0 summary score corresponding to a change in SGRQ total score equal to the MID of 4 points (Jones 2005). We also aimed to perform a receiver operating characteristic (ROC) curve to determine the change in WHODAS 2.0 summary score with equal/closest sensitivity and specificity to discriminate between those who improved in SGRQ total score beyond the MID and those who did not. Furthermore, the mean change in the WHODAS 2.0 summary score was calculated for participants who reported a perceived change in disability of at least ‘slightly better’ after rehabilitation, according to the global change rating scale (Revicki et al. 2008).

For the distribution‐based approach, the moderate effect size was calculated as half the standard deviation of the change in WHODAS summary score, and the absolute value of the minimal detectable change (MDC), based on the standard error of measurement (SEM), was calculated according to the equations below:

– Standard error of measurement (SEM) = SD of the pre‐rehabilitation WHODAS 2.0 summary score *x* $\sqrt{1-\text{intraclass}\;\text{correlation}\;\text{coefficient}\;}$
– Absolute value of the MDC at 95% (MDC_95%_): 1.96 × √2 × SEM

The analyses were carried out using SPSS version 22.0 (IBM, Armonk, NY, USA), and a *p*‐value < 0.05 was identified as statistically significant. GraphPad Prism 8 (GraphPad Software, La Jolla, CA, USA) was used to create the figure.

## 3 Results

### 3.1 Sample Characteristics

Forty individuals were assessed at baseline, but seven had to be excluded due to characteristics such as poor cognition, non‐adherence to the rehabilitation protocol, or no programme completion. There were no significant differences between included and excluded participants in age (59 ± 17 vs. 62 ± 17 years; *p* = 0.68), proportion of women (67% vs. 71%; *p* = 0.59), or body mass index (27.73 ± 7.62 vs. 23.55 ± 5.21 kg/m^2^; *p* = 0.18). Therefore, 33 individuals were included (Table 1). Their mean age was 59 ± 17 years, 67% were female, and 61% had a COPD diagnosis. The mean 6‐min step count (6MSC) was 85 ± 27 steps. In addition, the mean baseline SGRQ total score and WHODAS 2.0 summary score were 56.1 ± 11.9 and 44.98 ± 12.80, respectively. There were no missing data for the WHODAS 2.0 summary score, SGRQ scores, or the global rating of change at either the pre‐ or post‐rehabilitation assessments.

**TABLE 1:** Baseline characteristics of individuals with chronic respiratory conditions (*n* = 33).

Characteristic	*n*	Value
Age, years	33	59 ± 17
Female, *n* (%)	33	22 (67)
BMI, kg/m^2^	33	27.73 ± 7.62
Lung function	31	
FEV1, % predicted		52 ± 22
FVC, % predicted		69 ± 22
FEV1/FVC		0.61 ± 0.19
Respiratory health condition, *n* (%)	33	
COPD		20 (61)
Post‐COVID‐19 condition		8 (24)
Bronchiectasis		2 (6)
Tuberculosis sequelae		2 (6)
Asthma		2 (6)
Shrunken lung syndrome		1 (3)
Age‐adjusted Charlson index	33	3.6 ± 2.7
mMRC scale, *n* (%)	33	
0		2 (6)
1 or 2		13 (39)
3 or 4		18 (55)
6MSC	31	
Absolute value		85 ± 27
% of predicted		61 ± 17
SGRQ	33	
Symptoms		49.5 ± 17.5
Activities		77.8 ± 13.2
Impact		45.5 ± 15.8
Total score		56.1 ± 11.9
WHODAS 2.0 summary score	33	44.98 ± 12.80

*Note:* Data presented as absolute and relative frequency, or mean ± standard deviation.

Abbreviations: 6MSC: 6‐min step count; BMI: body mass index; COPD: chronic obstructive pulmonary disease; FEV_1_: forced expiratory volume in 1 s; FVC: forced vital capacity; FEV_1_/FVC: relationship between FEV_1_ and FVC; mMRC: modified Medical Research Council; SGRQ: Saint George's Respiratory Questionnaire.

### 3.2 WHODAS 2.0 Responsiveness to Rehabilitation

**FIGURE 1:**
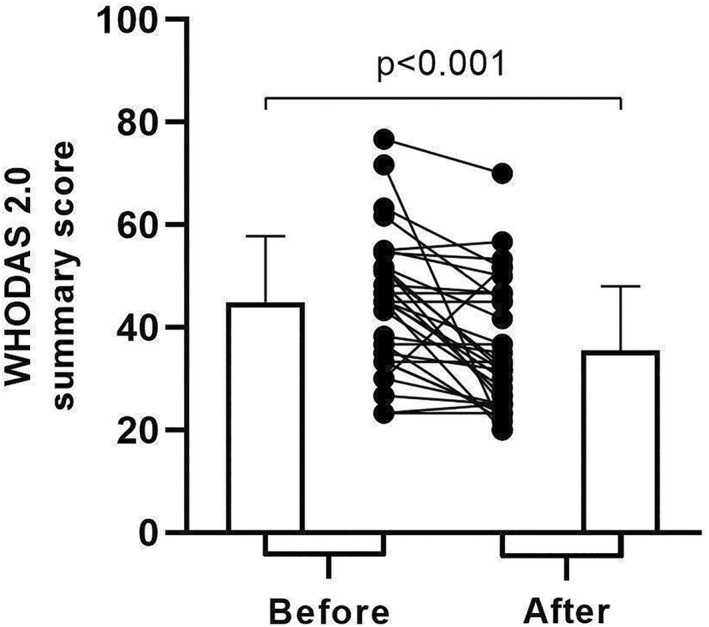
WHODAS 2.0 summary score in individuals with chronic respiratory conditions before and after the rehabilitation programme (*n* = 33).

There was a reduction (i.e., improvement) in the mean WHODAS 2.0 summary score from 44.98 ± 12.80 before rehabilitation to 35.49 ± 12.51 after rehabilitation (*p* < 0.001; Figure 1), with a mean difference of −9.49 ± 12.44 (95% CI −13.90 to −5.08). This mean difference represents an average reduction of 18 ± 26% from baseline. Twenty‐five individuals (76%) showed a reduction in the summary score (i.e., reduction in disability), while five individuals (15%) showed no change, and three (9%) showed an increase (i.e., worsening of disability). A reduction in SGRQ total score was also observed (56.1 ± 11.9 vs. 40.7 ± 19.1, respectively; *p* < 0.001), with an average reduction of −15.5 (95% CI ‐21.7 to −9.7). Twenty‐four individuals (73%) achieved a MID of 4 points for the SGRQ. The reduction in WHODAS 2.0 summary score was greater among those who showed a clinically important improvement in SGRQ total score compared to those who did not (−12.7 ± 11.9 vs. −1.0 ± 9.8, respectively; *p* = 0.02). As a secondary result, an increase in 6MSC was also observed from before to after rehabilitation (85 ± 27 vs. 115 ± 44 steps, respectively; *p* < 0.001).

### 3.3 WHODAS 2.0 Interpretability After Rehabilitation

The correlation between the change in WHODAS 2.0 summary score and the change in SGRQ total score was *r*_s_ = 0.36 (*p* = 0.04). The MID estimate for WHODAS 2.0 from the linear regression model was −5.53, which corresponds to the SGRQ's MID (≤ −4 points) (Jones 2005). The estimate from the ROC curve using SGRQ's MID as a reference was −5.00. Twenty‐two individuals (67%) stated that their disability was ‘better’ after rehabilitation, while 11 individuals (33%) reported it to be ‘slightly better’, with an average change in WHODAS 2.0 summary score of −5.50 (95% CI ‐13.04 to 2.04). Half SD of the change in WHODAS summary score was −6.22, while the SEM and MDC_95%_ values were −1.81 and −5.02, respectively (all values presented with a negative sign to facilitate interpretation).

## 4 Discussion

The main finding of this study is that WHODAS 2.0 was responsive to a rehabilitation programme incorporating physical exercise in individuals with chronic respiratory conditions, thereby confirming our hypothesis. The estimated MID for the WHODAS 2.0 summary score ranged from −6.22 to −5.02. To our knowledge, this is the first study to demonstrate the responsiveness of WHODAS 2.0 to a rehabilitation programme in this patient population and to provide MID estimates for this instrument.

Following rehabilitation, the WHODAS 2.0 summary score decreased significantly (indicating improvement), from 44.98 ± 12.80 before rehabilitation to 35.49 ± 12.51 after rehabilitation, demonstrating the instrument's responsiveness. In one of the few studies to assess WHODAS before and after a rehabilitation programme in individuals with chronic respiratory disease—specifically patients with COPD and depression—Alexopoulos et al. (2006) reported a significant improvement in WHODAS II summary score (a precursor to WHODAS 2.0), from 36.06 ± 7.45 to 30.52 ± 7.97, supporting these findings. Similarly, in other chronic disease populations, such as individuals with rheumatoid arthritis, WHODAS has also shown responsiveness following rehabilitation, with a significant reduction of −4.6 (95% CI −8.1 to −1.2) (Meesters et al. 2010).

Analysis of the interpretability of WHODAS 2.0 after rehabilitation showed that the MID ranged from −6.22 to −5.02. For researchers or clinicians wishing to apply a single MID value, −6.22 may be adopted as a more conservative estimate. Using the 12‐item WHODAS 2.0 in patients with chronic musculoskeletal pain, one study reported MID values between −4.7 and −3.1 (Katajapuu et al. 2020). However, as the summary score in that study was calculated as a simple sum (range 0–48), these values correspond to −9.79 and −6.46 on a 0‐100 scale (Katajapuu et al. 2020), which are broadly comparable with our findings. A limitation of that study was the exclusive use of distribution‐based methods to estimate the MID. In critically ill patients assessed 6 months after intensive care unit admission, Higgins et al. (2021) proposed a 10% reduction in the WHODAS 2.0 summary score as the MID, indicating a clinically meaningful improvement in disability. In the present study, the most conservative MID estimate (−6.22) corresponds to an approximate 14% reduction, with differences between studies likely reflecting variations in the characteristics of the populations studied.

This study has some limitations. One is the use of the 12‐item version of WHODAS 2.0, which does not provide domain‐specific scores. This version was selected because of its ease of administration compared with the 36‐item version, with the aim of using an instrument more likely to be adopted in clinical practice. Another important limitation was the small sample size with limited diagnostic diversity, which precluded subgroup analyses and may have reduced the statistical power and generalisability of the findings. In addition, the absence of a control group may have limited the interpretation of the results. Future studies should address these limitations and examine the influence of other factors, such as comorbidities (Mesquita et al. 2016).

In summary, WHODAS 2.0 was responsive to a rehabilitation programme incorporating physical exercise in individuals with chronic respiratory conditions, with a MID of at least −6.22. These findings should be interpreted in light of the study's limitations. This study supports the broader use of WHODAS 2.0 in both research and clinical practice, as one of the few generic disability measures developed within a biopsychosocial framework.

### 4.1 Implications of Physiotherapy Practice

Responsiveness plays an important role in clinical practice, as even small improvements or deteriorations can influence clinical decision‐making. WHODAS 2.0, a standardised and easy‐to‐use instrument developed by the WHO, demonstrated the positive impact of rehabilitation on disability in people with chronic respiratory conditions. A reduction of at least 6.22 may therefore be used as a threshold to identify clinically meaningful changes following intervention. In addition, WHODAS 2.0 is grounded in a biopsychosocial model and aligned with ICF codes, enhancing its robustness for clinical use. This is particularly relevant given that previously used instruments in rehabilitation studies of chronic cardiopulmonary diseases do not comprehensively capture ICF components (Stucki et al. 2007; E. A. Lima et al. 2024).

## Author Contributions

**Chayenne Chylld César Lopes:** conceptualization, methodology, validation, formal analysis, investigation, data curation, writing – original draft, writing – review and editing, visualization. **Vanessa Garcia de Lima:** validation, investigation, data curation, writing – review and editing. **Magno F. Formiga:** validation, investigation, data curation, writing – review and editing, supervision. **Rafael Mesquita:** conceptualization, methodology, validation, formal analysis, investigation, resources, data curation, writing – original draft, writing – review and editing, supervision, project administration. All authors provided final approval of the version to be submitted.

## Funding

This research did not receive any specific grant from funding agencies in the public, commercial, or not‐for‐profit sectors. There was only a personal grant, Vanessa Garcia de Lima was supported by a Master of Science grant from the Fundação Cearense de Apoio ao Desenvolvimento Científico e Tecnológico (FUNCAP), and from the Coordenação de Aperfeiçoamento de Pessoal de Nível Superior (CAPES).

## Ethics Statement

The study was approved by the Research Ethics Committee of the Walter Cantídio University Hospital on 16 November 2020 (approval number: 4.400.114).

## Consent

Written consent was obtained from all participants prior to data collection.

## Conflicts of Interest

The authors declare no conflicts of interest.

## Permission to Reproduce Material From Other Sources

The authors have nothing to report.

## Study Registration

The authors have nothing to report.

## Data Availability

The data that support the findings of this study are available on request from the corresponding author. The data are not publicly available due to privacy or ethical restrictions.
